# MEDICARE IS THE LAST HOLDOUT: EXAMINING THE IMPACT OF MEDICARE POLICY ON OLDER ADULTS’ ACCESS TO MENTAL HEALTH CARE

**DOI:** 10.1093/geroni/igz038.2547

**Published:** 2019-11-08

**Authors:** Matthew C Fullen, Jonathan Wiley, Amy Morgan, Gerard Lawson, Jyotsana Sharma

**Affiliations:** Virginia Tech, Blacksburg, Virginia, United States

## Abstract

Medicare is the primary insurance provider for approximately 59 million Americans, and the number of beneficiaries is expected to surpass 80 million by 2030. Currently, Medicare regulations allow psychiatrists, psychologists, clinical social workers, and psychiatric nurses to provide mental health services. These providers were last updated in 1989 with passage of the Omnibus Budget Reconciliation Act of 1989. Since that time, the mental health marketplace has changed dramatically, and Medicare beneficiaries are unable to access care from approximately 200,000 graduate-level mental health professionals with similar training to eligible Medicare providers. There is evidence that this Medicare mental health coverage gap (MMHCG) impacts both providers and beneficiaries. For example, some beneficiaries may begin treatment only to have services interrupted, or stopped altogether, once the provider is no longer able to be reimbursed by Medicare. We surveyed 6,550 members of the American Counseling Association, including 3,815 who identified themselves as practicing counselors. These individuals work in diverse contexts (e.g., community mental health agencies, private practice, and integrated care settings). Survey results indicated that a significant number of practicing counselors turn away or refer Medicare beneficiaries who seek mental health care due to the MMHCG. In-depth interviews were also completed with eight licensed mental health professionals who detailed the challenges they and their clients experienced. Participants perceived a discrepancy between Medicare’s intended aims to promote health and provider restrictions that were confusing and frustrating to navigate. Participants concluded that the MMHCG has a negative impact on older adult clients.

